# Psychometric validation of the Reported and Intended Behaviour Scale (RIBS) in Hungary with a particular focus on ‘Don’t know’ responses and further scoring recommendations

**DOI:** 10.1186/s12889-023-16707-3

**Published:** 2023-09-12

**Authors:** D. Őri, E. Vass, K. Vajsz, K. Vincze, V. Sztancsik, A. Szemán-Nagy, L. Simon

**Affiliations:** 1 Institute of Behavioural Sciences, Semmelweis University, Nagyvárad Tér 4., 1089 Budapest, Hungary; 2 Department of Mental Health, Heim Pál National Pediatric Institute, Budapest, Hungary; 3 Department of Psychology, Illinois Institute of Technology, Chicago, USA; 4 Department of Psychiatry and Psychotherapy, Semmelweis University, Budapest, Hungary; 5 Institute of Psychology, Eötvös Loránd University, Budapest, Hungary; 6 Department of Clinical Psychology, Semmelweis University, Budapest, Hungary; 7 Institute of Environmental Medicine, Karolinska Institute, Solna, Sweden; 8 Department of Psychiatry, Clinical Center of the University of Debrecen, Debrecen, Hungary; 9 Department of Personality and Clinical Psychology, University of Debrecen, Debrecen, Hungary

**Keywords:** Reported and Intended Behaviour Scale, Psychometric validation, Mental health stigma scale, Discriminatory behavior, Scoring of don't know answers

## Abstract

**Aims:**

Reported and Intended Behaviour Scale (RIBS) was designed to measure mental health stigma-related behaviors in the general public. We aimed to examine its psychometric properties and validate the scale in a Hungarian non-clinical community sample. The secondary aim of this study was to assess the appropriateness of the current scoring recommendations of ‘Don’t know’ responses being coded as neutral, which had never been investigated before. In addition, we provide an overview of the results of already existing studies on the scale.

**Methods:**

Hungarian participants completed the RIBS within this cross-sectional online survey study and were considered non-clinical individuals based on a cut-off point of the Global Severity Index T score of 63 on the Symptom Checklist-90-Revised. Confirmatory factor analysis, reliability measures, and comparative analyses were performed.

**Results:**

Of the *n* = 5,701, *n* = 5,141 participants were included in the analysis. The mean age was 27.8 ± 11.1 years, and 89.2% (*n* = 4,587) of the sample were female. The unidimensional structure was supported by good model fit indices (RMSEA = 0.031, CFI = 0.999, TLI = 0.996, and WRMR = 0.006). Internal consistency of the RIBS and its test–retest reliability with a 5-month follow-up period were found to be good (Cronbach’s alpha = 0.88 and ICC = 0.838). We found statistically significant differences between the total scores when the ‘Don’t know’ responders were excluded from the sample or when they were coded as neutral as recommended by the scale authors (16 (IQR:13–18) vs. 15 (IQR:13–18) *p* < 0.0001). There were also statistically significant differences between ‘Neither agree nor disagree’ and ‘Don’t know’ participants in several aspects of lived experiences of mental health problems.

**Conclusions:**

The RIBS demonstrated good psychometric properties and can be transferred to the Hungarian context. It will be a valuable tool in assessing stigmatizing behavior and testing the efficacy of antistigma programs. Our results suggest that ‘Neither agree nor disagree’ and ‘Don’t know’ responses bear different meanings, and coding should account for this.

## Background

People with mental illness and those who use psychiatric services have long been stigmatized, which impairs their quality of life. Six percent of the world’s population live with a severe mental illness that measurably decreases their quality of life [1]. A good quality of life has two pillars: a safe, independent home and a decent job opportunity. The stigmatization of mental illness worsens the chances of affected people finding a job or housing [2–5]. A person living with a mental illness may seem strange to the public: sad or awkward, irritable or irritating. Others seem extremely shy, distrustful, or unexpectedly intrusive [6]. The attitude and reaction of others depend on their prior knowledge, personality, and the actual situation and condition in which they meet the other, as well as the social and cultural traditions that lead them to act, react, or neglect [1, 7].

Although mental illness stigma has been defined to include components of knowledge, attitudes, and behavior, only a few psychometrically tested instruments assess behavioral discrimination [8]. Self-reported discriminatory behavior is limited and presumed, or intended behaviors are less often measured than attitudes [9, 10]. The Reported and Intended Behaviour Scale (RIBS) is an instrument based on the Star Social Distance Scale to assess reported (present and past) and intended (future) behavioral discrimination against people with mental health problems in the general population [11]. The RIBS is the only validated questionnaire that analyzes the presence of reported and intended stigmatizing/discriminatory behaviors against people with mental health problems in the general population [12].

The RIBS was developed in the United Kingdom as a brief and feasible instrument consisting of two parts: reported or actual behaviors and intended behaviors assessed in four different contexts: (1) living with, (2) working with, (3) living nearby and (4) continuing a relationship with a person with a mental health problem [8]. The scale items and response options are listed in Table 2. As the first part is designed to assess the prevalence of these behaviors, psychometric studies mainly focus on the intended behavior part, which has a unidimensional factor structure [8]. The internal consistency for the intended behavior items ranged between 0.75 and 0.95 assessed in various populations, including adolescents, in the following countries: the United Kingdom [8, 11], Japan [13], China [14], Italy [12], the Czech Republic [15], Uganda, Sweden [16], Catalonia [17], New Zealand [18], France [19], Brazil [20], Ukraine [21], Columbia [22], Ghana and Kenya [23]. Cronbach’s alpha values for the whole scale were between 0.65 and 0.79 in the aforementioned Czech [15], in Libanese [24] and in Indian studies [25] and in Macedonian, Turkish, Azeri, Kazah and Polish medical students [26].

The scoring system of the scale appears to be easy to follow, the second part is scored on a 5-point Likert scale, and both contain a ‘Don’t know’ (DK) response option. Several questions arise when the optimal number of answer choices are discussed, such as whether an even or an odd number of response alternatives is ideal. When a middle response alternative is provided, people may choose the midpoint to minimize cognitive costs (satisficing)’, or simply due to fatigue or poor motivation to complete the survey, or it might mean a socially desirable hidden DK [27, 28]. Adding the DK alternative makes it possible to distinguish between true neutral opinion holders and those who do not hold an opinion on the issue. Thus, participants are neither forced to choose among the answer choices nor exposed to the selection of middle-alternative rather than explicitly admitting their ignorance by selecting or volunteering DK. Therefore, providing the DK option appears to be beneficial in reallocating ‘face-saving don’t knows’ from the mid-point to the DK category, which significantly alters descriptive and multivariate inferences [28]. In contrast with the benefits, adding a DK option tends to increase nonresponses, does not improve data quality, and prevents respondents from sharing potentially meaningful opinions, particularly those held with lower confidence [29]. As for DK answer applicability, the results appear to be mixed in the existing literature. The meta-analyses of the amount of random measurement error correlate in numerous survey items, including DK or ‘No-opinion’ responses resulting in significantly fewer random errors compared to the omission of these answers [30]. However, there is evidence of the contrary [31]. Moreover, offering DK options had no significant impact on reliability or validity [32].

There are various guidelines in the literature for evaluating DK responses, including coding them as neutral on a Likert scale, omitting these respondents from the entire sample, and scoring them as missing data that could be replaced by various methods such as mean, median or mode, regression imputation, maximum likelihood or multiple imputation methods [33]. It should be noted that we cannot eliminate potential biases if we decide to replace these data; however, ignoring these responses is likely to reduce the effective sample size, yielding reduced statistical power as well [29, 33]. If scale designers decide to offer a DK option, it is beneficial to obtain substantive data from respondents who opted for this answer choice by asking them a follow-up question, for example, whether they tend to choose one of the substantive response options [34]. The RIBS authors suggested scoring the DK answer as neutral on the Likert scale; however, this does not allow separating the DKs from the neutral answers, which would be beneficial. Albeit their solution does not lead to the reduction of the effective sample size; as the scale authors do not provide the respondents with a follow-up question and recommend using 3 points, we fail to get appropriate information from these two separate response categories. It should be highlighted that none of the above-mentioned psychometric studies had specifically examined the suitability of scoring the DK answers, nor did they investigate the possible differences between the subsample of those who chose DK and ‘Neither agree nor disagree’ (NAND) answer choices for the scorable part of the RIBS.

### Aims

Our primary aim was to examine the psychometric properties of the RIBS, validate the scale in a Hungarian non-clinical community sample, and examine the psychometric results in contrast to those in other countries. The secondary aim of this study was to assess the appropriateness of the existing scoring recommendations of DK responses being coded as neutral. We finally aimed to examine the differences between the lived experiences of groups who chose NAND and DK answers.

## Methods

### Study overview

This was a cross-sectional study that used an anonymous online survey to measure the stigmatizing attitudes towards people with mental illness in a non-clinical population within the framework of the Hungarian National Antistigma Program. The research team contacted the participants by e-mail, and the survey link was also distributed on social media platforms as part of a campaign through the network of the Deep Breath Project (Hungarian psychoeducational media content provider presented on various platforms) and the Hungarian Association for Behavioural and Cognitive Therapies. Additionally, forwarding the link of the survey was also an option to the targeted population; therefore, the convenience sampling method contained snowball sampling techniques as well. The questionnaire package included the Symptom Checklist-90-Revised (SCL-90-R) [35], a 90-item questionnaire used to assess psychological problems, whose Hungarian adaptation was found to be a valid and reliable measurement in Hungary [36]. As the aim of the study was to reach people who did not suffer from any mental illness, according to the design of the study and the guidelines of the SCL-90-R, participants were considered to be non-clinical individuals based on a cut-off point of the Global Severity Index T scores of 63 (219 points on SCL-90-R) [37]. This decision was primarily driven by the need to minimize potential confounding factors that could influence the results. By focusing on a non-clinical sample, we aimed to explore attitudes and behaviors in an everyday context, without the potential influence of formally diagnosed and treated mental health conditions. This could lead to the exclusion of those who may not be aware of their mental health problems, possibly indicating undiagnosed and untreated conditions and vice-versa, respondents above the cut-off should not be considered a formal 'clinical' population due to the lack of formal diagnoses. The study was completed with a repeated-measure segment, where a small proportion of the respondents (*n* = 17) completed the target questionnaire for a second time using code words for identification to allow the investigation of temporal reliability.

### RIBS scoring

The first four items calculate the frequencies of ‘reported or actual behaviors’ among participants who are not necessarily engaged in those behaviors. Therefore, these items are not scored. Items 5 to 8 indicate ‘intended behaviors’. These are scored on a Likert scale from 1 to 5 points depending on the level of agreement of the respondent. Strong disagreement with a statement scores 1 point, while strong agreement scores 5 points. The total score is calculated by adding the response values of items 5 to 8. The scale developers suggest coding the DK answer choice as neutral (i.e., 3 points).

### Scale translation

English version of the RIBS was first translated into Hungarian by an experienced English–Hungarian clinical psychologist translator, and then translated back into English by a bilingual English-Hungarian qualified medical interpreter according to INDIGO guidelines [38]. An iterative procedure was used to resolve the discrepancies between the original and the back-translated versions of the scale.

The following sociodemographic details were collected from the participants: age, gender; place of residence, marital status, lived experience of mental health problems, including possible knowledge of friends and first- or second-degree relatives affected by any mental health problems.

### Statistical analyses

Demographic data points are expressed as sample size (n) and percentage (%). To describe the age of the participants, mean scores and standard deviations were used. Where the data did not follow a normal distribution, the median and interquartile ranges were used. Confirmatory factor analysis (CFA) was performed to examine the model fit. CFA was performed using diagonally weighted least squares (WLSMV) method, which is specifically designed for ordinal data as the variables are nonparametric. To evaluate the model fit, we calculated the following indices and adopted the generally recommended criteria: chi-square (χ2), root mean square error of approximation (RMSEA, < 0.06), comparative fit index (CFI, > 0.95), Tucker-Lewis Index (TLI, > 0.95) [39]. Standardized model estimates, their standard eror (SE), *p*-values are provided along with the communalities. To measure internal consistency, Cronbach’s alpha coefficients were calculated for the whole scale, and in cases where an item was deleted (0.70–0.95) [40]. To describe the test–retest reliability, the intraclass correlation coefficient (ICC) was calculated along with the 95% confidence intervals based on a mean-rating (k = 2), absolute-agreement, two-way mixed-effects model (ICC < 0.50 poor, 0.50–0.75 moderate, 0.75–0.90 good, > 0.90 excellent) [41]. The Mann–Whitney U test was used to compare the two groups, the Kruskal–Wallis test to more than two groups, whereas the chi-square test was applied to analyze the ratio of personal affection and sociodemographic data with different scorings of DK answers. Effect sizes were measured by eta-squared (η^2^) (η^2^ = 0.01 small, 0.06 medium, 0.14 large effect) [42] and Cramer’s V (V for df 1 0: no association between the variables, 0.1 small, 0.3 medium, 0.5 large effect) [43]. We used IBM SPSS 25 (Apache Software Foundation, USA), MPlus 6.12 (Muthen and Muthen, USA), and SAS OnDemand 9.4 (SAS Institute Inc., USA) software for the analyses.

## Results

### Participants

A total of 5,701 people completed the online survey. To obtain a non-clinical sample, the SCL-90-R’s Global Severity Index T score of 63 was used as cut-off, resulting in a total of 5,141 participants. Participant characteristics are shown in Table 1.

**Table 1:** Participant characteristics

Participant characteristics	Mean and SD
Age	27.8 ± 11.1
	**n (%)**
Sex	
Male	524 (10.2)
Female	4,587 (89.2)
Place of living	
Lives in Hungary	4,684 (91.1)
Capital	2,064 (40.1)
County Capital	981 (19.1)
City	383 (7.4)
Town	1,014 (19.7)
Village	699 (13.6)
Marital status	
Living with a partner/married	2,540 (49.4)
Single	2,601 (50.6)
Lived experience	
Have a family member with a mental health problem	1,930 (37.5)
Have a friend with a mental health problem	2,973 (57.8)
Have ever lived with someone with a mental health problem	1,265 (24.6)
Have ever been treated for mental health problem	1,045 (20.3)
Have ever attended psychotherapy	1,689 (32.9)

The vast majority (89.2%) of the participants were female; half of them were married or in a relationship. More than half of them (57.8%) had a friend who struggled with mental health problems. More than one in each three had a family member with mental health problems. Approximately one in five participants had been treated for some mental health problem.

### Distribution of the responses

Overall, participants appeared to use the full range of response options (see Table 2), although responses *skewed toward* a higher level of agreement with the given statements, indicating a potential ceiling effect. The frequency of not knowing the level of agreement ranged from 13.0% to 40.9% for the first four items of the questionnaire and from 4.4% to 12.1% for the second part of it.

**Table 2:** Response frequencies for the RIBS

		Yes n (%)	No n (%)	Don’t know n (%)			
1	Are you currently living with, or have you ever lived with someone with a mental health problem?	1,722 (33.5)	2,749 (53.5)	670 (13.0)			
2	Are you currently working with, or have you ever worked with someone with a mental health problem?	1,368 (26.6)	2,352 (45.7)	1,421 (26.6)			
3	Do you currently have, or have you ever had a neighbour with a mental health problem?	833 (16.2)	2,205 (42.9)	2,103 (40.9)			
4	Do you currently have, or have you ever had a close friend with a mental health problem?	3,005 (58.5)	1,229 (23.9)	907 (17.6)			
		Disagree strongly n (%)	Disagree slightly n (%)	Neither agree nor disagree n (%)	Agree slightly n (%)	Agree strongly n (%)	Don’t know n (%)
5	In the future, I would be willing to live with someone with a mental health problem	349 (6.8)	506 (9.8)	1,436 (27.9)	1,331 (25.9)	899 (17.5)	620 (12.1)
6	In the future, I would be willing to work with someone with a mental health problem	198 (3.9)	270 (5.3)	960 (18.7)	2,024 (39.4)	1,425 (27.7)	264 (5.1)
7	In the future, I would be willing to live nearby to someone with a mental health problem	234 (4.6)	297 (5.8)	954 (18.6)	1,895 (36.9)	1,459 (28.4)	302 (5.9)
8	In the future, I would be willing to continue a relationship with a friend who developed a mental health problem	163 (3.2)	145 (2.8)	744 (14.5)	1,885 (36.7)	1,980 (38.5)	224 (4.4)

### Results of the confirmatory factor analysis

Only the second four items of the scale should be included in the factor analysis; the scale showed a unidimensional structure on which all items loaded appropriately. The fit indices were: Chi2 = 10.553, RMSEA = 0.031 95%CI(0.015–0.051), CFI = 0.999, TLI = 0.996, and WRMR = 0.006. For the model results that include the standardized estimates, standard errors, the corresponding *p*-values, along with the communalitities, please see Table 3.

**Table 3:** Standardized model results

F1 BY	Estimate	SE	*p*-value	Communality
RIBS5	0.751	0.009	0.000	0.563
RIBS6	0.865	0.008	0.000	0.748
RIBS7	0.824	0.009	0.000	0.680
RIBS8	0.808	0.009	0.000	0.654

*SE* standard error

### Internal consistency

Cronbach's alpha value for the second part of the scale ‘intended behavior subscale’ was 0.884, which is considered ‘good’ according to the ranges defined by Cronbach. As presented in Table 4, item reduction did not lead to an increase in Cronbach's alpha. It should be noted that Cronbach’s alpha values were higher when DK answers were excluded from the sample than when coded as neutral (3 points) and pooled with NAND responses.

**Table 4:** Internal consistency of the RIBS by using two different scorings for don’t know responses

	Don’t know answers are excluded from the sample (*n* = 4,317)	Coded don’t know as neutral (*n* = 5,141)
Cronbach’s alpha total scale	0.884	0.875
RIBS items	Cronbach’s alpha if an item deleted	
In the future, I would be willing to live with someone with a mental health problem	0.871	0.857
In the future, I would be willing to work with someone with a mental health problem	0.835	0.824
In the future, I would be willing to live nearby to someone with a mental health problem	0.847	0.838
In the future, I would be willing to continue a relationship with a friend who developed a mental health problem	0.850	0.841

### Test–retest reliability

**Fig. 1:**
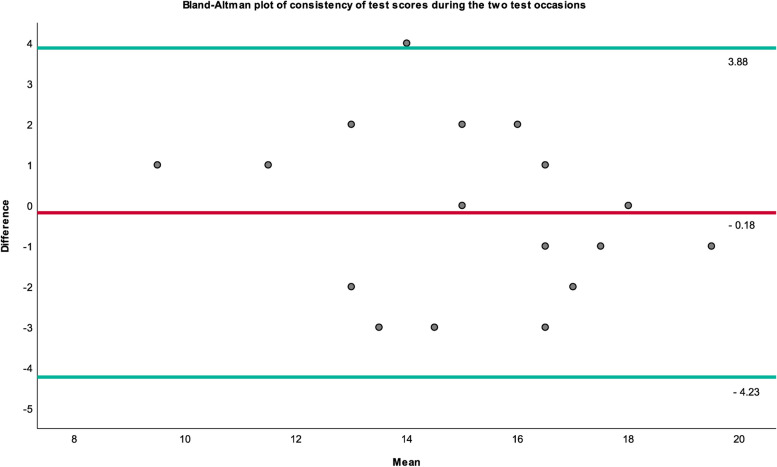
Bland Altman plot of consistency of test scores

In order to calculate test–retest reliability, a subsample of subjects (*n* = 17) completed the survey twice with a follow-up of five months (median 153 [23–297] days). The Bland and Altman plot demonstrated a good level of agreement, with no proportional bias (t = -0.971, *p* = 0.347) (See Fig. 1). The ICC was 0.838 95%CI (0.548–0.942) for the intended behaviors of the RIBS, indicating good to moderate test–retest reliability.

### Differences based on different scorings of don’t know answer choices

For the second part of the scale, a total of 824 people chose the DK option for at least one item, and 93 of them indicated not knowing the answer for all four items. The median total scores were statistically significantly higher when the DK responders were excluded from the sample than when they were coded as neutral (16 (IQR: 13–18) vs 15 [13–18] *p* < 0.0001).

There were no statistically significant differences between Hungarian men and women in terms of the number of DK answers (16.4% of Hungarian men and 16.1% of women answered DK for at least one statement of RIBS second part question, Chi-square = 2.34, *p* = 0.31, η^2^ = 0.0002). With regard to education, significant differences were found, where the DK answers were the highest among those who graduated from vocational school (at least one DK answer: 27.7%, at least 3 DK answers: 5.56%) (Chi-square = 41.06, *p* < 0.0001, η^2^ = 0.007). As for age, a significant difference was also supported. However, no age group emerged from the sample based on the frequency of DK answers (Chi-square: 126.2, *p* < 0.0001, η^2^ = 0.024).

Similar to DK answers, no significant gender differences were found in choosing NAND (Chi-square = 2.07, *p* = 0.35, η^2^ = 0.0002). Age and education differences were also consistent with the findings of DK answers (Age: Chi-square = 94.66 *p* = 0.014, η^2^ = 0.018, Education: Chi-square = 13.57, *p* = 0.01, η^2^ = 0.002). Table 5 shows statistically significant differences in several aspects of lived experiences of mental health problems between individuals who chose NAND or DK for at least for one item and those who did not choose either option.

**Table 5:** Differences between neutral and don’t know responses are based on the lived experience of the participant

RIBS item	Type of lived experience	‘Neither agree nor disagree’ respondents (n)	‘Do not know’ respondents (n)	χ^2^	*p*-value	Cramer’s V
In the future, I would be willing to live with someone with a mental health problem	Have a family member with a mental health problem (*n* = 1,930)	512 (27%)	159 (8%)	23.56	< 0.0001	0.133
	Do not have a family member with a mental health problem (*n* = 1,546)	424 (27%)	237 (15%)			
	Have a friend with a mental health problem (*n* = 2,973)	791 (27%)	274 (9%)	10.54	0.0012	0.085
	Do not have a friend with a mental health problem (*n* = 896)	267 (30%)	139 (16%)			
	Have ever lived with someone with a mental health problem (*n* = 1,265)	320 (25%)	81 (6%)	23.45	< 0.0001	0.107
	Have never lived with someone with a mental health problem (*n* = 3,876)	1,116 (29%)	539 (14%)			
In the future, I would be willing to work with someone with a mental health problem	Work with people with mental health problem (*n* = 864)	158 (18%)	24 (3%)	8.88	0.0029	0.085
	Do not work with people with mental health problem (*n* = 4,277)	802 (19%)	240 (6%)			
	Have a friend with a mental health problem (*n* = 2,973)	488 (16%)	104 (3%)	5.41	0.0201	0.080
	Do not have a friend with a mental health problem (*n* = 896)	191 (21%)	62 (7%)			
	Have ever lived with someone with a mental health problem (*n* = 1,265)	215 (17%)	36 (3%)	9.75	0.0018	0.089
	Have never lived with someone with a mental health problem (*n* = 3,876)	745 (19%)	228 (6%)			
In the future, I would be willing to live nearby to someone with a mental health problem	Work with people with mental health problem (*n* = 864)	170 (20%)	39 (5%)	3.98	0.0460	0.056
	Do not work with people with mental health problem (*n* = 4,277)	784 (18%)	263 (6%)			
In the future, I would be willing to continue a relationship with a friend who developed a mental health problem	Have ever lived with someone with a mental health problem (*n* = 1,265)	158 (12%)	32 (3%)	5.27	0.0217	0.074
	Have never lived with someone with a mental health problem (*n* = 3,876)	586 (15%)	192 (5%)			

Only those types of lived experiences are displayed next to the RIBS5-8 items, where we found differences between those participants who choose DK and NAND for the given RIBS item by using chi-square test. The percentage shown is calculated by dividing the frequency of the response option (choosing at least one DK or NAND) by the total number of people with the characteristic (e.g. having a family member with mental illness)

## Discussion

We tested the feasibility and reliability of the RIBS on a large non-clinical sample in Hungary and evaluated several aspects relating to the reliability and validity of the scale. We specifically focused on the differences between participants who answered NAND and DK in the second part of the scale. As the RIBS had not yet been used in Hungary before, weprovide evidence for the psychometric properties of the scale, particularly in terms of test–retest reliability, internal consistency, and goodness of model fit in this country. In addition, we provide more profound insights into the middle point answer choices and challenge the scoring recommendations.

In summary, our results support the good psychometric properties of the RIBS scale. Overall Cronbach’s alpha was 0.87–0.88, suggesting robust internal consistency, regardless of the way of coding the DK answers. Additionally, the confirmatory factor analysis focusing on the intended behavior scale verified a unidimensional structure) as well as good to moderate test–retest reliability, further enhancing the consistency of literature. Table 6 provides an overview of the investigated samples and the results of the factor analysis of the international studies carried out so far, including our results. The RMSEA varied on a wide range, while the incremental fit indices were excellent in each psychometric study. The overall best model fit was detected in Italy [12], followed by the current Hungarian study results, indicating excellent model fit. The RMSEA was 0.06 in the United Kingdom [11] on an adolescent sample and 0.07 in Brazil [20] as well as in Japan [13], which are considered borderline fit. The RMSEA in the French [19] and Columbian [22] studies was higher than the acceptable range. Notably, the scale author did not include CFA results in their article about the development of the RIBS [8].

**Table 6:** Overview of the results of the factor analyses on the RIBS in international studies

Research group, year	Investigated population	Method of estimation		Results		Country
			RMSEA	CFI (TLI)	GFI (AGFI)	
Yamaguchi et al., 2014 [13]	undergraduate and postgraduate students *n* = 224	not reported	0.072	0.955	0.956 (0.916)	Japan
Pingani et al., 2016 [12]	general public *n* = 447	not reported	0.023	0.994	0.987 (0.975)	Italy
Garcia et al., 2017 [19]	nursing students *n* = 268	not reported	0.092	0.985 (0.954)	-	France
Mansfield et al., 2020 [11]	Adolescents 11–15 years *n* = 1032	not reported	0.06	1 (1)	-	United Kingdom
Ribeiro et al., 2021 [20]	Community sample (caregivers) *n* = 1357	WLSMV	0.07	1 (1)	-	Brazil
Campo-Arias et al., 2021 [22]	Adolescents 10–17 years *n* = 350	not reported	0.17	0.97 (0.92)	-	Columbia
Current study	Non-clinical population *n* = 5141	WLSMV	0.031	0.999 (0.996)	-	Hungary

*AGFI* Adjusted Goodness of Fit, *CFI* comparative fit index, *GFI* Goodness of Fit, *RMSEA* root mean square error of approximation, *TLI* Tucker-Lewis Index, *WLSMV* diagonally weighted least squares

It is important to mention that the median total scores were significantly higher when we excluded the DK responders from the sample than coded them as neutral (16 (IQR: 13–18) vs 15 [13–18] *p* < 0.0001). Although this observation does not necessarily have an impact on the results of the examination of psychometric properties, it can most likely influence the results and the interpretation of the scale when used in a clinical trial. Hence, we do not suggest associating these answers with the value of ‘3’ or labeling them as ‘neutral’, as it would mean confusion of holding a neutral attitude and refusing to answer due to other possible reasons. In terms of motivation, respondents giving DK answers and those who tend to provide NAND with answers were found to have more experience (e.g., living or working with) with people suffering from mental health issues, they were more prone to be neutral, and their answer was more likely to be NAND. This explanation is consistent with our earlier claims on the DK answers, as they also allow the respondent not to have any experience with mental illnesses. In addition to experience, it is worth examining the potential role of sociodemographic factors.

As results show, no differences can be supported between those who are more prone to give DK answers and NAND along the sociodemographic characteristics. Differences were found only in the amount of personal experience with mental health issues, although the effect sizes were small. Nevertheless, the resultsemphasize the relevance of differentiating between the two mentioned response types.

The RIBS aims to assess stigma-related behaviors towards people with mental health problems in general and does not differentiate between diagnostic classes. While there is a growing body of literature on stigma associated with different mental health conditions, it is important to note that the extent and nature of stigma may vary across diagnostic classes. Research has shown that certain mental health conditions, such as schizophrenia and bipolar disorder, tend to be highly stigmatized compared to other diagnoses like depression and anxiety disorders [44]. Schizophrenia is often associated with misconceptions, fear, and stereotypes perpetuated by the media and society at large that individuals with schizophrenia are labeled as dangerous or unpredictable [45], so as are people with alcohol or drug dependence [46]. On the other hand, conditions such as depression and anxiety disorders may be relatively less stigmatized due to their higher prevalence and increased public awareness campaigns [47]. Nonetheless, individuals with these conditions may still encounter stigmatization. The stigma towards different diagnostic classes is mainly measured by case vignettes in the literature. The Mental Health Knowledge Schedule [48] assesses the public's comprehension of mental health and records whether various conditions, such as depression and stress etc., are viewed as mental disorders by the respondent. Although there are tools designed specifically for various conditions, such as depression [49] or suicide stigma [50], currently, there is a lack of stigma measures that assess stigma towards different conditions in a single measure. Future research is needed to gain a more comprehensive understanding of differences in stigma across major diagnostic classes, and the scientific community would benefit from a single measure that examines different components of stigma and across different conditions. The RIBS test can be classified as one which aims to measure the stigma toward people with mental health problems; however, it focuses explicitly on the behavioral aspect rather than capturing the underlying attitudes and beliefs that drive stigmatizing behaviors. Although the RIBS does not distinguish between specific diagnoses, which may result in a limited understanding of condition-specific stigmatization, generalist approaches like the RIBS offer the advantage of allowing an overall perspective of how the general public views or intends to behave towards individuals with mental health problems. This provides an opportunity to compare the viewpoints of communities across different countries and cultures, as various diagnoses may have unique aspects of the stigma associated with them due to societal perceptions and cultural factors.

In addition to examining the psychometric properties of the RIBS scale, our results may provide information about the level of stigma in Hungary, for which to date, only one scale, the Opening Minds Stigma Scale for Healthcare Providers, is available and validated, which is designed to measure the attitudes of health care workers [51, 52]. Highlighting the limitation of non-representativeness of each study for their countries, the Hungarian study population reported higher levels of contact with people suffering from psychiatric disorders in comparison with the subjects of English [8], Italian [12], Czech [15], and Japanese [13] studies.

As for the willingness to have future contact with people with mental illness, Hungarian responders showed less willingness only in comparison with English responders. If we take a closer look at the data, it can be seen that the relationship between more personal experience and willingness is not linear. In this context, the lowest ‘willingness’ scores are usually explained by the recent start of the deinstitutionalization process in the relevant countries. However, Hungarian results appear to contradict this explanation, as in this country, this process has barely started [12]. Another probable interpretation of such discrepancy could be based on the nature of antistigma campaigns. Campaigns in Hungary usually focus on the facilitation of contacts between the clinical and non-clinical population, whereas in other places (e.g., Italy) the focus is on the transfer of relevant and accurate information to the general public, or in other countries (e.g., England) both aspects are emphasized [53, 54]. Although these approaches may provide a satisfactory explanation for the results in general, Hungary appears to be still out of line. This country does not have a national-level antistigma program, and the efficacy of local campaigns may vary on a wide scale. Hence, we assume that the characteristics of the sample might have a significant effect on the results. Female responders and people living in the capital city were overrepresented in the sample. It also has to be highlighted that most responders were recruited as part of an antistigma campaign and through the network of the Deep Breath Project and the Hungarian Association for Behavioural and Cognitive Therapies, which could have narrowed the range of responders to those interested in the topic that has a potential impact on the representativeness of the sample and generalizability of the findings. Furthermore, as these results were found in a non-clinical sample (SCL-90-R Global Severity Index T scores of 63), future research should investigate clinical populations to gain deeper insights into the experiences and attitudes of people with diagnosed mental health problems. Our findings can serve as a basis for such investigations, helping to bridge the gap between non-clinical and clinical populations in understanding stigmatising attitudes and behaviours.

Although this study presents robust data on the Hungarian population, limitations should be mentioned. As previously discussed, female city dwellers were overrepresented in the study due to the nature of the recruitment process; hence, our sample cannot be considered nationally representative. Another significant issue is the small sample size in the test–retest reliability evaluation. We could identify two main reasons behind this limitation. First, the responders usually did not remember the code word; thus, we could not match the test–retest surveys in many cases. Second, responders forgot to complete the questionnaires for the second time. To address these limitations, we plan to use an electronic data capture system (e.g., REDCap) in future research, which allows us to avoid using code words and sends frequent reminders to the responders.

In summary, our results are in line with previous literature in terms of providing further evidence that RIBS is a useful and reliable assessment tool to measure stigmatizing behavior in the non-clinical population. Furthermore, as the first validated assessment tool in this regard in Hungary, the RIBS might be an essential component in future research, as it enables measuring the efficacy of existing antistigma programs in our country and thus can aid the development of more effective approaches to fighting mental health stigma.

## Conclusions

In this study, the RIBS was translated into Hungarian, and this translation was validated in a sample of non-clinical participants. Although only minor differences were found in psychometric properties based on different coding patterns, our results suggest that neither agree nor disagree (NAND) and ‘don’t know’ (DK) responses bear different meanings, and coding should account for this. Our findings do demonstrate that the RIBS can be transferred to the Hungarian context. The interplay between lived experiences of participants and their differences in choosing NAND or DK responses to the statements has the potential to inform further research and deepen our understanding of processes involved in stigmatization and thus aid the development of antistigma interventions.

## Data Availability

The datasets used and analysed during the current study are available from the corresponding author (DŐ) on reasonable request. The data are not publicly available as the stigmatizing attitudes toward people with mental health problems of the large sample of the Hungarian general population is not yet published.

## Abbreviations

χ^2^: Chi-square
η^2^: Eta-squared
AGFI: Adjusted goodness of fit,
CFA: Confirmatory factor analysis
CFI: Comparative fit index
df: Degree of freedom
DK: Don’t know
GFI: Goodness of fit
ICC: Intraclass correlation coefficient
IQR: Interquartile range
NAND: Neither agree nor disagree
RIBS: Reported and Intended Behaviour Scale
RMSEA: Root mean square error of approximation
SCL-90-R: Symptom Checklist-90-Revised
SE: Standard error
TLI: Tucker-Lewis Index
WLSMV: Diagonally weighted least squares method
